# An Investigation on the Sampling Frequency of the Upper-Limb Force Myographic Signals

**DOI:** 10.3390/s19112432

**Published:** 2019-05-28

**Authors:** Zhen Gang Xiao, Carlo Menon

**Affiliations:** Schools of Mechatronics Systems Engineering and Engineering Science, Simon Fraser University, 250-13450 102 Avenue, Surrey, BC V3T 0A3, Canada; zgx@sfu.ca

**Keywords:** force myography, FMG, muscle, wearable, motions, human–computer interface, signal sampling

## Abstract

Force myography (FMG) is an emerging method to register muscle activity of a limb using force sensors for human–machine interface and movement monitoring applications. Despite its newly gained popularity among researchers, many of its fundamental characteristics remain to be investigated. The aim of this study is to identify the minimum sampling frequency needed for recording upper-limb FMG signals without sacrificing signal integrity. Twelve healthy volunteers participated in an experiment in which they were instructed to perform rapid hand actions with FMG signals being recorded from the wrist and the bulk region of the forearm. The FMG signals were sampled at 1 kHz with a 16-bit resolution data acquisition device. We downsampled the signals with frequencies ranging from 1 Hz to 500 Hz to examine the discrepancies between the original signals and the downsampled ones. Based on the results, we suggest that FMG signals from the forearm and wrist should be collected with minimum sampling frequencies of 54 Hz and 58 Hz for deciphering isometric actions, and 70 Hz and 84 Hz for deciphering dynamic actions. This fundamental work provides insight into minimum requirements for sampling FMG signals such that the data content of such signals is not compromised.

## 1. Introduction

Force myography (FMG) is an emerging technique to register muscle activity of a limb [1]. This technique utilizes multiple force sensors to detect the pressure variation on the surface of the limb during movements [2]. Such variations can be correlated to muscle contraction levels and, therefore, can be used to estimate different limb movements for physical activity monitoring or human–machine interface applications [1]. For example, FMG signals from near the wrist or around the proximal forearm have been used to detect the number of grasping actions during a pick-and-place exercise [3] and to control an external robotic device such as a hand prosthesis [4]. Compared to other methods which also can decipher limb action from muscles (e.g., surface electromyography), FMG has better signal stability against environmental factors such as the change in skin humidity, change in skin temperature, and power line interference [5]. Also, its signal is less expensive to extract as it requires less complicated hardware for signal conditioning [5].

The term “FMG” first appeared in a publication by Wininger et al. in 2008 [2], but the idea of using force transducers to detect muscle movements dates back to as early as the 1960s [6]. The same technique was also referred to as “residual kinetic imaging” or “muscle pressure distribution mapping” by different researchers [7,8]. To avoid confusion, the term “FMG” is used throughout this paper to represent all techniques which involve the use of force sensors to detect pressure variations of a limb. Despite the newly-gained popularity of FMG among academic researchers, there is still a lack of study on its fundamental characteristics. One commonly-asked question about the FMG technique is how fast the FMG signal should be sampled. FMG researchers have used different sampling frequencies in their publications. The FMG sampling frequencies reported in the literature from 2006 to 2018 are listed in Table 1.

The studies shown in Table 1 have used different sampling frequencies ranging from 6 Hz to 1 kHz, however, an in-depth explanation of the rationale for selecting such frequencies is not provided. In most cases, the sampling frequency was dictated by the hardware available for the study. Obviously, when there are no constraints on power consumption, size, computational resources, and cost, FMG signals can be sampled with a very high sampling rate such that no information is lost. However, it is different in practice. For instance, the easy-to-use aspect of FMG makes it suitable for wearable portable applications. To extract and process the signal, wearable platforms often rely on an energy-efficient microcontroller which has lower computational power than a typical processor in a PC; hence, there is a need to identify the suitable sampling frequency for wearable real-time FMG applications. In this context, the suitable sampling frequency is defined as the minimum sampling frequency, capable of capturing the signal characteristics.

FMG detects the patterns of muscle movements related to different actions or static gestures. When only static gestures are of interest, the sampling frequency does not need to be high (e.g., <10 Hz), as the FMG pattern does not alter drastically once the gesture is stabilised [10]. However, during activities of daily living (ADL), we need to perform complex actions in isometric or dynamic conditions in which a higher sampling frequency is required. An isometric condition describes a scenario in which the force is applied to a target but results in no obvious displacement. Hand squeezing without an object is an example of an isometric action in which the forearm muscles are contracted, but there is no obvious displacement of the hand. For such an action, the FMG signal pattern is mostly tied to the change of muscle contraction levels. According to the investigation by Freund et al., the frequency of change of a forearm muscle contraction level can be as high as 1 kg per 100 ms [41]. Therefore, capturing the full action characteristics cannot be guaranteed if the FMG signals are sampled at 10 Hz. On the other hand, a dynamic condition describes a scenario in which the force applied to a target results in a displacement. Every displacement of the limb is a result of dynamic actions. For such an action, not only do the FMG signals relate to the change of muscle contraction level, but they are also subjected to motion artifacts. To capture all the information needed, FMG signals may need to be sampled at an even higher frequency. In addition to different types of actions, the location of the FMG sensors (i.e., FMG extracted from the distal end of the forearm versus the proximal end) may also contribute to different sampling frequency requirements.

To find out the suitable sampling frequency for FMG applications, we conducted an experiment to examine FMG signals during different rapid movements in both isometric and dynamic scenarios. The aim of this study is to identify the minimum sampling frequency needed for recording upper-limb FMG signals without sacrificing signal integrity. Such results provide guidelines on how to sample FMG signals for various human–machine interfaces and activity monitoring applications.

## 2. Method

An experiment was designed to collect high-quality FMG data from healthy participants during rapid upper limb actions in order to identify the minimum sampling frequencies that are appropriate for human–machine interfaces and activity monitoring applications. The obtained data would be downsampled with different frequencies to investigate the discrepancy between the original signal patterns and the downsampled ones. Also, the power density of the FMG signal patterns would be studied to gain insight into the signal characteristics needed to identify the minimum frequencies.

The Office of Research Ethics at Simon Fraser University approved the protocol of this research. Twelve volunteers gave informed consent to participate in the study (6 males and 6 females aged from 21 to 41 years old). The experimental setup, the protocol, and the data analysis procedures are presented in the following subsections.

### 2.1. Experimental Setup

**Figure 1:**
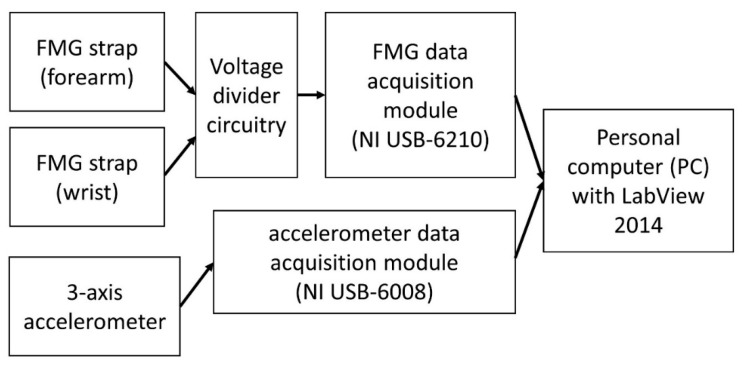
Schematic of the experimental setup.

The schematic of the experimental setup is shown in Figure 1. The setup includes two FMG straps, a three-axis analog accelerometer, a voltage divider circuit, two data acquisition modules, and a personal computer.

**Figure 2:**
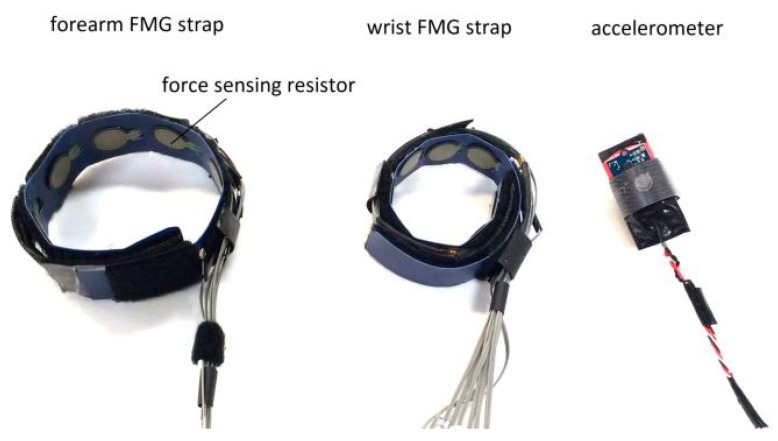
FMG and motion sensing devices.

The two FMG straps (see Figure 2) were designed to capture FMG signals from both the proximal and the distal end of the forearm. Each strap has eight force sensing resistors (FSR402, Interlink Electronics, Inc., Los Angeles, CA, USA) embedded inside to capture the pressure from the contact region between the surface of the limb and the sensors. Each FSR has a force sensitivity range of 0.1 to 10.0 N and a device rise time that is less than 3 µs [42]. These properties ensure that the sensor can pick up the minuscule changes in FMG signals. The signals from these sensors were extracted using an array of voltage divider circuitries and converted into digital data using a data acquisition (DAQ) device with a 16-bit analog-to-digital resolution (USB-6210, National Instruments Inc., Austin, TX, USA). The voltage divider circuit has one base resistor per channel and the values of these base resistors affect the output response of the FSR sensors. In this study, the base resistors were set to 5 kOhm to ensure a linear FSR voltage reading response within the actuation force range of FMG applications (i.e., <1.2 N for FSR402) [43]. For such a configuration, the sensitivity of the FSR sensor is approximately 2.3 V/N [43].

The accelerometer (see the right-hand side of Figure 2) was designed to be affixed on the index finger to capture the data during dynamic movements. The data from this accelerometer were used to obtain reference information about the performed movements. The analog signals of the accelerometer were converted into digital data using another DAQ (USB-6008, National Instruments Inc., Austin, TX, USA).

The signals of both the FMG straps and the accelerometer were sampled at 1 kHz with the DAQs and were recorded using custom software developed using LabView 2014 from National Instruments Inc.

### 2.2. Experimental Procedure

**Figure 3:**
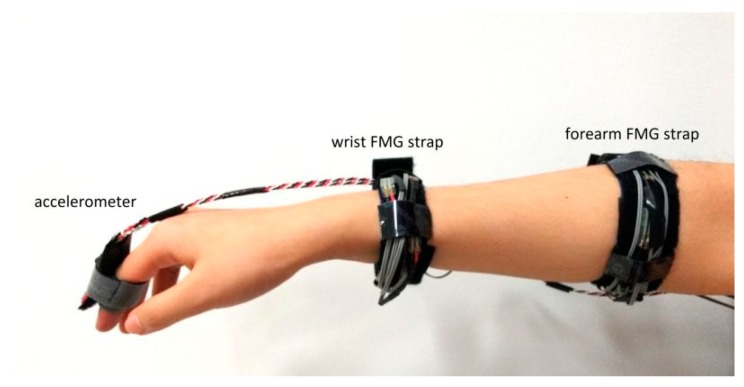
Sensor placement.

Before starting the data collection, a research assistant explained the objective of the experiment to each participant, and the participant provided consent before proceeding to the next step. The accelerometer and the FMG straps were then donned by the participant with the help of the research assistant. Note that the accelerometer was worn on the index finger only during dynamic actions. An example of the placement of the FMG straps is shown in Figure 3. The distal forearm strap was placed right behind the head of the ulna, and the proximal FMG strap was placed on the bulk region of the forearm which has the maximum cross-sectional circumference. For simplicity, hereinafter, we refer to the distal FMG strap as the wrist FMG strap and to the proximal forearm strap as the forearm FMG strap.

The core of the experiment was to capture the FMG signal patterns during the fastest upper limb actions used in daily activities (i.e., finger and wrist actions). By capturing the FMG patterns associated with the fastest upper limb movements, we ensured the validity of our results for all possible actions that can occur during ADL, which cover scenarios that require either normal speed or rapid movements. Two types of movements were considered for the study, isometric and dynamic movements. Specifically, three isometric actions and four dynamic actions were selected: hand squeezing, palm pressing, index finger pressing, index finger tapping, padding, shaking, and simulating a drumming action. These actions were selected as they are common actions used in ADL [44,45,46,47], and cover a broad range of possible finger and wrist movement combinations. For instance, hand squeezing activates the same muscle group as the power grasp, which is one of the most important actions we use in daily activities [45,47]. Palm pressing, which uses the same muscle group for wrist flexion-extension, is also a fundamental action used in daily activities and a key action included in many FMG investigations [9,20,23,48,49]. FMG was shown to be able to estimate finger movement status [22,33]; therefore, index finger pressing was included in the protocol to represent this fine finger action. Index finger pressing is another commonly-used action in daily activities such as mouse clicking [50]. Index finger tapping was selected as the dynamic version of the finger pressing action. Padding, shaking, and drumming actions were selected to represent rapid wrist flexion–extension, pronation–supination, and abduction–adduction actions, respectively.

**Figure 4:**
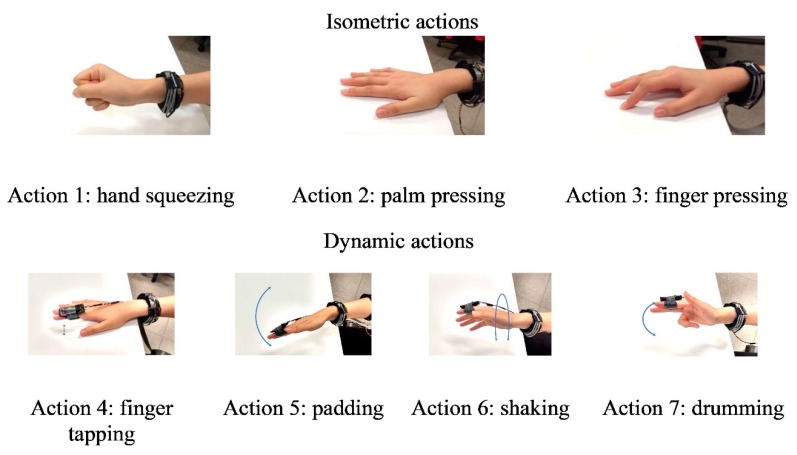
Selected hand actions.

During the experiment, participants were instructed to perform the actions shown in Figure 4 as fast as possible for 5 seconds each with their dominant hand. Only the action of the dominant hand was considered as it was expected to produce actions at a faster speed than the non-dominant one. Once completed, they repeated all actions two more times for a total of three trials. For isometric movements (Actions 1–3), participants were asked to apply roughly 10% of the maximum voluntary contraction force. For dynamic movements (Actions 4–7), participants were asked to move within 10% of the full range of motion. Both the maximum voluntary contraction force and the full range of motion were assessed prior to data collection with a hand dynamometer and a protractor. However, 10% of the maximum voluntary contraction force and range of motion were used as a guide only and were not monitored during the test. Participants were allowed to exceed the 10% limit. The purpose of such a step was to ensure that participants did not over fatigue the hand during the test and allowed them to achieve the fastest natural movement possible.

For the squeezing action, participants were asked to hold the hand in mid-air with a 90° elbow angle while making a fist. When the data collection started, participants quickly squeezed the hand with a mild force as described above and then relax the muscles as fast as they could control. They would repeat the action until the data collection stopped. For the palm pressing action, they rested the hand on the table with the wrist hanging in mid-air and the elbow bent at a 90° angle. For this task, it was important for participants to hold the elbow at 90° to ensure the force generated was mainly from the forearm muscles and not from the bicep or triceps muscles from the upper arm. During data collection, they quickly pressed the palm against the table mildly without making any obvious movements and then relaxed as fast as possible. The finger pressing action scheme was similar to the one of the palm pressing action, but instead, only the index finger was applying force against the table. The finger tapping action scheme was similar to the finger pressing action, with the exception that the index finger was moving up and down as quickly as possible and an accelerometer was attached to the finger tip to monitor the action. For the three remaining actions, participants were once again holding the hand in mid-air with the elbow bent at a 90° angle. For the padding action, participants flexed and extended the hand as quickly as possible while keeping the fingers straight. For the shaking action, participants shook the hand by supinating and pronating the wrist joint as quickly as possible while keeping the hand in a relaxed state. For the drumming action, they moved the hand by abducting and adducting the wrist joint as quickly as possible while keeping the hand in a gun-like gesture.

### 2.3. Data Processing and Result Analysis Procedure

Once data collection was completed, each FMG dataset from participants was first normalised according to its full range of amplitude variations from the signal reading. In this normalisation scheme, raw signals were first subtracted from their minimum values and were then divided by the full range of the dataset. Such a step reduced the inherited bias due to the participants’ physical differences and the different tightness of the straps during the donning procedure.

Next, for every single trial of the repeated actions, the normalised signals were downsampled with step sizes ranging from 2 to 1000 samples, which corresponds to sub-sampling frequencies of 500 Hz to 1 Hz. In order to have a quantitative measure of the difference between the original signals and the downsampled ones, we needed to obtain the same number of data points from the resampled FMG signals which were achieved by using the linear interpolation method. The difference between the two versions of signal patterns was represented by the mean of the root-mean-square-error (RMSE) of the 8 FMG channels. The equation for the mean RMSE is shown as

(1) $$avg.\;RMSE=\;\frac{\sum_{j}^{m}\sqrt{\frac{\sum_{i}^{n}{\left(x_{ij}-{x^{\prime }}_{ij}\right)}^{2}}{n}}}{m}$$

where x is the original sample value with a sample index, i, and a channel index j; x’ is the interpolated value from a downsampled signal with the corresponding indexes; m is the number of FMG channels; and n is the number of samples. To improve readability, we refer to the mean of RMSE of the eight channels as RMSE from now on. Once the RMSE values from all participants’ trials were computed, we analysed the obtained values separately based on four categories. The four categories were: (1) isometric actions with forearm FMG, (2) isometric actions with wrist FMG, (3) dynamic actions with forearm FMG, and (4) dynamic actions with wrist FMG.

Then, various factors contributing to the different RMSE values for the entire dataset were analysed by using N-way ANOVA with Tukey’s Honestly Significant Difference Procedure (provided in the statistics and machine learning toolbox of MATLAB 2017a). The factors considered for this analysis were the range of different resampling frequencies, action type, signal type, hidden factor associated with participants’ data, and choice of different interpolation methods. Due to the amount of complexity of the N-way ANOVA test required and the limited computational power available, we only focused on the resampling frequencies that were from 10 Hz to 100 Hz. We further divided this set of frequencies into nine groups based on an incremental frequency range with a step of 10 Hz. The action types were the isometric actions and the dynamic actions, and the signal types were the types of FMG extracted from the forearm and wrist. Finally, the choices of interpolation methods were the linear, cubic, and spline interpolations.

In order to gain insight into the power of the FMG signals, we computed the power spectral density (PSD) using the fast Fourier transform (FFT) for each of the eight unfiltered FMG signals that were collected during each of the 5-second actions. The maximum PSD reading of the eight FMG channels for each frequency component within the entire spectrum was then defined as the maximum FMG power density. This maximum power density represented the highest possible power which could be captured using the eight FSR sensors. Note that the FFT assumes the periodic nature of the signals, which is the case for this specific experimental protocol. However, when FMG is used for predicting limb actions in daily activities, such an assumption may not be valid. Therefore, we also computed the PSD using the autoregressive (AR) method, which does not assume the periodic nature of the signals, to provide another investigation on the power of the signals. The PSD calculated using the AR method is included in the Appendix A.

## 3. Results

In this section, we present an example of the FMG signals captured for different actions to provide a visual representation of the FMG patterns, followed by the analysis of the RMSE between the original signals pattern and the ones with different downsample frequencies. Then, the results on the different factors contributing to the difference in RMSE are provided. Finally, we investigate the PSD of the FMG patterns to gain an understanding of the FMG power characteristics.

**Figure 5:**
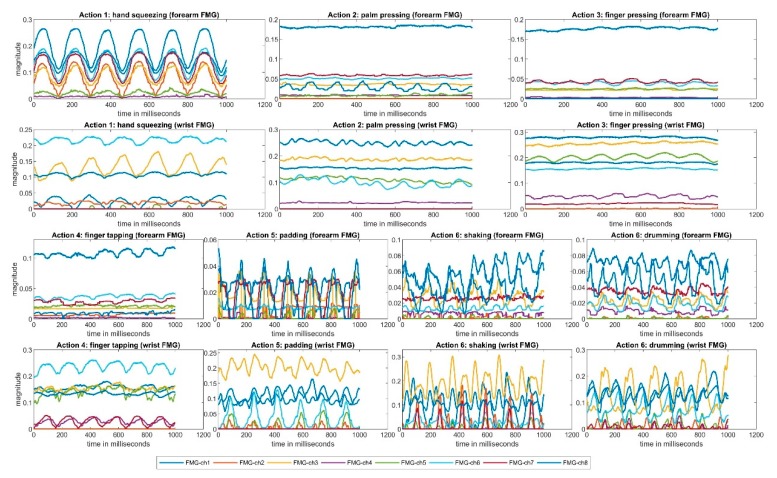
One second of FMG signals collected during one trial.

An example of a one-second period of collected FMG data from a participant during one repetition is shown in Figure 5. The *y*-axes show the normalised FMG magnitude and the *x*-axes indicate time in milliseconds. The forearm FMG are shown in the first and the third rows, while the wrist FMG are shown in the second and the fourth rows. The different colored lines indicate the different FMG channel readings. For each action, at least one repeated FMG pattern can be observed, suggesting that the FMG setup was capable of detecting the actions selected in this study.

**Figure 6:**
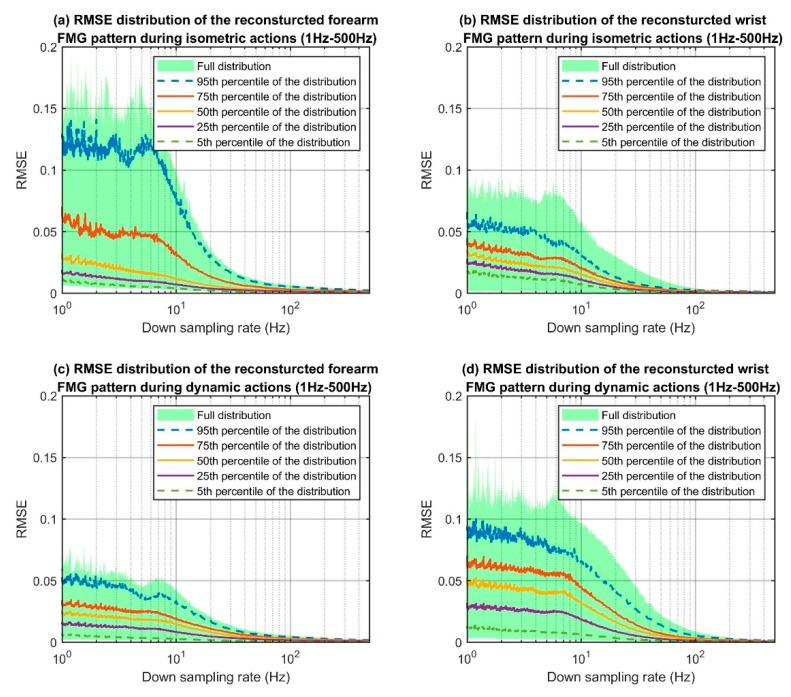
RMSE distributions of different resampling frequencies.

The RMSE value for each downsampled action sequence was calculated to form the RMSE distribution graphs that are shown in Figure 6. For each graph, the *x*-axis shows the frequencies ranging from 1 Hz to 500 Hz in log scale and the *y*-axis shows the corresponding RMSE values. To summarise, the entire dataset was obtained from 12 participants and they each performed the seven actions sequence three times. The seven actions included three isometric actions and four dynamic actions. Hence, for the isometric actions, there were 108 RMSE values per one downsampled signal pattern. For dynamic actions, there were 144 RMSE values per one downsampled signal pattern to form the distribution graph. The full distribution of the RMSE is shown as a light green background and different percentiles of the distribution (i.e., the 95th, 75th, 50th, 25th, and 5th percentiles), are indicated with different colored lines. For all categories, the RMSE values decreased as the sampling frequencies increased. Since the majority of the FMG literature used 100 Hz for experiments involving dynamic actions, we considered this sampling frequency as the point of reference for discussion. At a frequency of 100 Hz, the RMSEs of the 95th percentile curve dropped below 0.001 for all categories.

The results of the N-way ANOVA on RMSE distribution are partly shown in Table 2. With the exception of different interpolation methods, all of the selected factors have significant effects on the RMSE results using a significant level of *p*-value = 0.05. The most influential factor is the range of the resampling frequency as it has the largest F-value (F = 5560), followed by the action type (F = 308), the signal type (F = 75), and the participant factors (F = 54).

**Figure 7:**
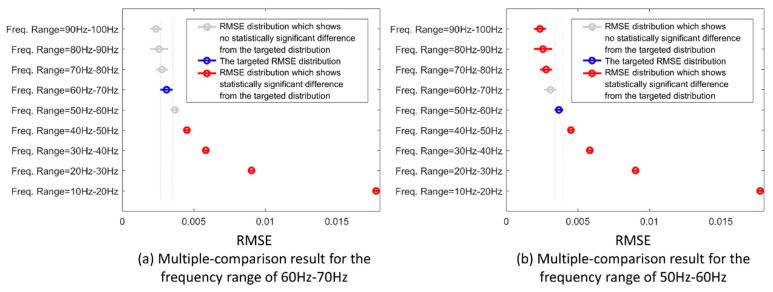
RMSE distributions for different resampling frequency ranges. The circle indicates the mean RMSE and the horizontal line shows the +/‒ one standard deviations.

The multiple-comparison test results for different frequency ranges are shown in Figure 7. The *x*-axis indicates the RMSE distribution for each group of the downsampled frequencies. The corresponding range for each group is indicated on the *y*-axis. The blue symbol indicates the targeted group which is being tested against the others. The gray color symbols indicate the corresponding groups that have no statistical difference against the targeted group; while the red color symbols indicate the corresponding groups that have a statistical difference against the targeted one.

Taking all factors into consideration, the RMSE distribution of the frequency range of 60–70 Hz was statistically different from the ones below 50 Hz, but did not differ from the ones with higher frequencies (see Figure 7a). In contrast, the RMSE distribution of the range of 50–60 Hz was different from the ones above 70 Hz, despite its similarity to the 60–70 Hz range (see Figure 7b). This result suggests that the minimum sampling frequency should be at least above 60 Hz for general FMG applications.

**Figure 8:**
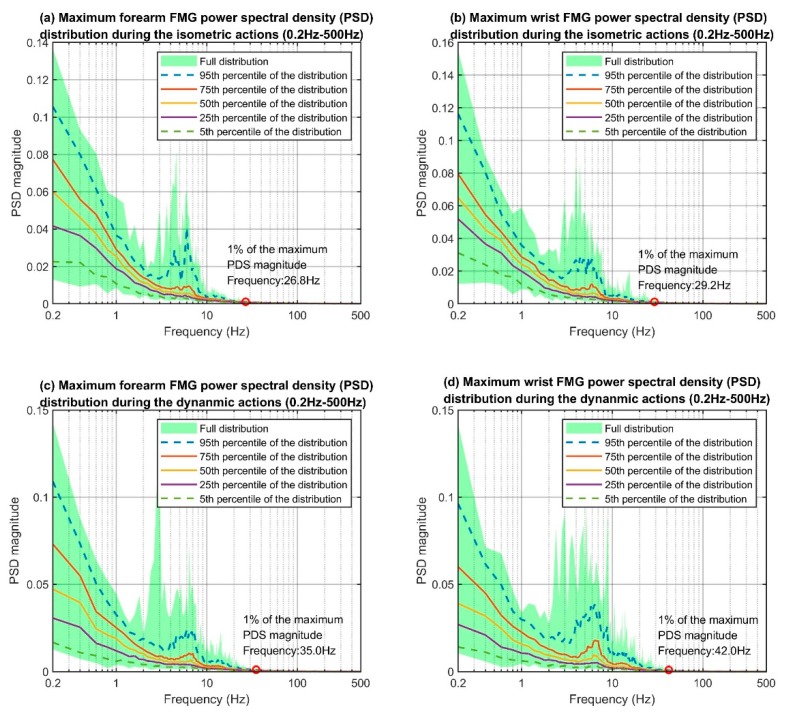
Maximum FMG power spectral density (PSD) distributions.

The maximum power spectral density distributions of each of the four data categories (i.e., the categories that were based on the combination of the two action types and two sensor placements), are shown in Figure 8. The *y*-axis of each plot indicates the PSD magnitudes and the *x*-axis indicates the frequency components from 0.2 Hz to 500 Hz. The lower bound of the FMG bandwidth should be 0 Hz, as the raw FMG signals were the output voltage from the voltage divider circuitry and were always positive. However, the magnitude of the 0 Hz component was disproportionally larger than the rest, therefore, it was ignored for the investigation. The overall trends in each category’s distribution are similar. The PSD magnitudes decrease as the frequency increases roughly to the 3 Hz mark. After that, the PSD magnitudes start to increase and reach the local maxima at around 5 Hz to 7 Hz, and finally resume the decreasing trends. There are no more peaks at the tail-end of the plots, which suggests that the FMG setup did not pick up any high-frequency noise such as the 50/60 Hz powerline interference.

**Figure 9:**
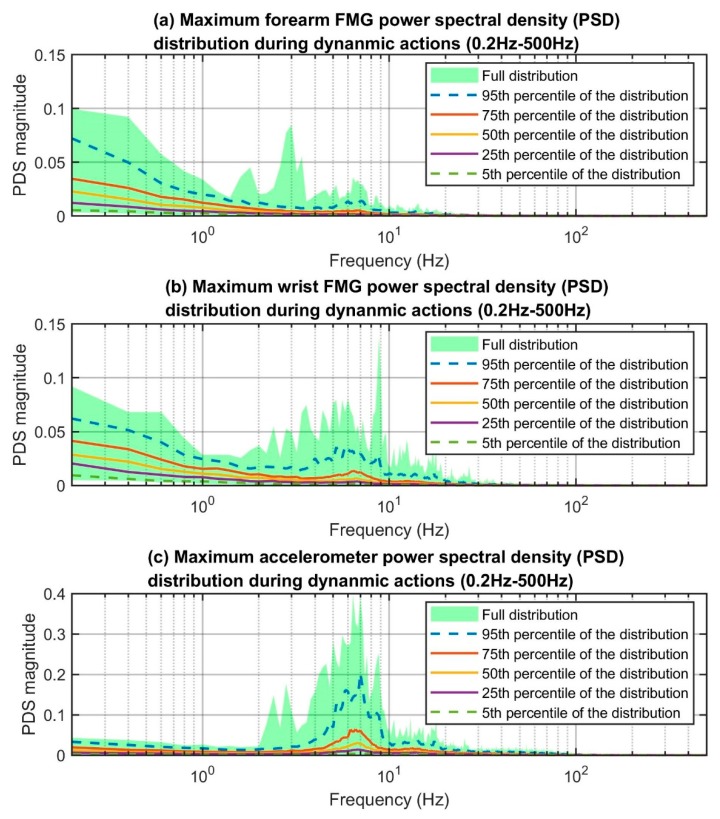
Maximum FMG power spectral density (PSD) distribution of FMG and the accelerometer signal during dynamic actions.

We considered the upper bound frequency of the FMG patterns to be the frequency associated with 1% of the maximum PSD reading on the 95th percentile distribution curve. The maximum PSD of all four groups resided at 0.2 Hz, but the 1% mark varied slightly across the different categories (see the red circles in Figure 8). The frequencies associated with the 1% marks are 26.8 Hz, 29.2 Hz, 35.0 Hz, and 42.0 Hz for the corresponding groups. Overall, the upper bound frequencies for dynamic actions were higher than those for isometric actions. This result was expected as the FMG sensor captured movement artifacts (i.e., sliding between the sensors and skin) during dynamic actions while such artifacts were less prominent in isometric scenarios. The upper bound frequencies between the forearm and wrist FMG were similar for the isometric actions (26.8 Hz vs. 29.2 Hz) but different in the dynamic scenarios (35.0 Hz vs. 42.0 Hz). The main reason could be the difference in sensor placement. The rapid movements were generated at the hand and fingers, which were much closer to the wrist than the forearm. The PSD distribution plots of the forearm, wrist, and the accelerometer data (see Figure 9) support such reasoning as the shape and position of the PSD of the accelerometer data are closer to those of the wrist than those of the forearm for the higher frequency portion.

## 4. Discussion

We considered 100 Hz as the frequency of interest for discussion since the majority of the FMG literature use the 100 Hz sampling frequency in their experiments involving dynamic movements. A sampling frequency of 100 Hz produced RMSEs less than 0.001 for all categories’ data and it was larger than double the highest upper bound frequency captured in the experiment. Therefore, based on the Nyquist theorem [51], this frequency could capture the majority of FMG signal characteristics without suffering from aliasing errors.

Five factors that could potentially affect the RMSE were investigated. The frequency range was an obvious factor that could affect the results, as shown in Table 2. Based on the results of the multiple comparison test, we established that the minimum sampling frequency for general FMG applications should be above 60 Hz, as the sampling range between 60–70 Hz did not significantly increase the RMSE when compared to a sampling frequency between 90–100 Hz. Furthermore, based on the PSD analysis, we suggest that a minimum sampling frequency of 84 Hz be used, especially when the application involves fast dynamic actions. Other than the frequency range, the action type was another important factor, as it had the largest F-value in the ANOVA test, which was about 4 times larger than the one following it (i.e. the signal type). This finding suggests that the minimum sampling frequency should be selected based on whether the application involves dynamic movements or not. Moreover, it is important to pay attention to the source of FMG (i.e., wrist or forearm) for the same action. Part of the information content that FMG signals carry is specific to each participant. To acknowledge this, the role of hidden factors in participants’ data on error was investigated, and it was found that such individual-specific data affects the RMSE. The choice of interpolation method was also investigated. However, it was found that the interpolation method by itself does not have a significant effect on the RMSE distribution. Interestingly, when considering different frequency ranges together, the interaction between the two factors did have a significant influence on the distribution (see the sixth row of Table 2). Based on the spread of the distribution, we expected that the choice of interpolation methods would dictate the RMSE value at the lower frequency range, but that its influence would become less as the frequency increases.

After the initial decrease in PSD plots (Figure 8), the magnitudes rise again to the peak at around the 5–7 Hz range and resume the decreasing trend at around the 10 Hz mark. This upward trend of magnitudes was mainly tied to how fast a healthy individual could move the hand voluntarily. This frequency range supports findings in the literature showing that the maximum speed for finger tapping is around 6.5 Hz [52,53]. FMG signal patterns represent movements of muscle groups and although each muscle can contract at a very fast speed [41], the overall manifestation of the movement is reduced at the surface due to different layers of muscles and skin. As a result, the dominant FMG signal content is more related to the speed at which the action is performed rather than muscle contraction.

FMG is often used along with different machine learning algorithms to decipher hand gestures and limb actions. For example, Jiang et al. were able to detect 48 different static gestures only using the raw FMG magnitudes with a support vector machine classifier [54]. However, in order to decipher dynamic gestures or limb actions, temporal features need to be extracted for machine learning purposes. A temporal feature is usually extracted from a processing window which contains multiple consecutive samples. For instance, the second derivative of an FMG signal is a temporal feature of the signal itself, which can be calculated from the difference in magnitude between two consecutive samples. This feature can be associated with the action speed and the change of instant position of the limb, which is of importance for the velocity control scheme of a prosthesis or other robotic devices. It is important to know that the numeric manifestation of a temporal feature highly depends on the sampling frequency of the signal. If the signal is sampled at a rate that is slower than what is required for the targeted action, the extracted temporal feature may not contain information that can be associated with the action. This scenario may lead to a sub-optimal prediction performance for a machine learning model. By sampling FMG signals above the suggested minimum frequency, it is guaranteed that the capability of FMG can be fully exploited for the subsequent processing step.

This study focused on investigating the FMG frequency associated with hand actions and did not include movements of the arm. Studies show that FMG on the forearm can detect arm movements as well [9], which means such movements might also affect the pattern of FMG signals. However, for the purpose of this study, it was sufficient to identify the frequency bandwidth associated with actions of the hand, which are faster than arm movements. Therefore, the sampling frequency obtained in this study is likely applicable for actions involving arm movements.

The action force used in the experiment was low in order to achieve the fastest action possible. The effect of different action force levels on the PSD was not investigated in this study. It is known that some action force (i.e., the grasping force) can be correlated with FMG using regression techniques [2]. The higher the variation in grasping force, the more distinct FMG patterns can be observed, and vice versa. If the manifestation of the grasping force is too small to be picked up by the FMG sensor, the overall spectral magnitude will be small, and the shape of the spectral graph will be flat. If the change in grasping force is large enough to be pickup by FMG sensors, then at least one distinct peak can be observed from the spectral graph. However, a larger grasping force will require more time for the muscle to generate and recover, hence, the dominant peaks of the spectral graph will be shifted towards a lower frequency. As long as the investigation includes the fastest action, the upper bound of the FMG frequency in the PSD distribution graph should not change. Therefore, a higher force level is not expected to change the results of this investigation.

Twelve healthy participants volunteered in this research, their main occupations were college students and university researchers, not professional athletes nor individuals that practice fast hand actions such as a professional drummer. For instance, the fastest drummer can achieve an average drumbeat of 16.8 beats per second per hand [55]. Such individuals can achieve much quicker actions than the general public, therefore, testing results may show different PSD distributions if such individuals participate in a study. A future study is warranted for sportsmen and individuals with these specific traits.

The sensors used in this investigation were the popular polymer thick film sensor (i.e., FSR402), which have been used in many FMG studies. Even though it has good force range and a quick response time which made it suitable for this investigation, it is not designed for precise force measurements and one should exert caution when using FSR for FMG applications. For instance, it has a good single part force repeatability error (i.e., +/−2%), but a high part-to-part force repeatability error of +/−6%. In order to reduce the discrepancies due to a different sensor, the sensor reading should be normalised before further processing. Another limitation of the FSR sensor is its nonlinear voltage output response when using the voltage divider circuit. The nonlinearity exaggerates when the actuation force on the sensor is high. However, when the actuation force is low, which is the case for FMG applications, the nonlinearity can be reduced by selecting the right base resistor [43]. For instance, by using a 5 kOhm base resistor in this study, the voltage response exhibits a strong linear trend within the 1.2 N of actuation force [43]. Also, FSR is known to have large hysteresis and long-term drift error, which are 10% and 5% respectively [42]. However, they are of lesser concern for FMG applications because of the low actuation force and the fact that the sensor does not experience constant large loads for a long period. A more precise force sensor would benefit FMG research.

## 5. Conclusions

This work focused on identifying the minimum sampling frequencies for FMG signals during rapid isometric and dynamic actions. The FMG signals were captured with a high sampling frequency of 1 kHz and were then reconstructed with different frequencies lower than the original one. We showed that the commonly used 100 Hz sampling frequency was sufficient to capture the majority of FMG characteristics as a low RMSE (i.e., less than 0.001) was obtained. Other than the frequency factor, different action types and signal types, and participants’ characteristics were all found to affect the RMSE. It was also found that the suitable sampling frequency could potentially be further reduced if the action and signal types are known beforehand. Based on Nyquist theorem, we suggested the minimum sampling frequencies of 54 Hz and 58 Hz for forearm and wrist FMG signals during isometric actions, and 70 Hz and 84 Hz for forearm and wrist FMG signals during dynamic actions. This study provides guidelines for FMG signals sampling, which is important for future research and development of FMG technology for human–machine systems.

## Figures and Tables

**Table 1:** FMG sampling frequency reported in the literature.

Action Type Involved	Sampling Frequency (Hz)	Reference
Static	1000	[9,10]
	100	[8]
	30	[11]
	50	[12]
	25	[13]
	20	[14,15]
	15	[16]
	10	[4,17,18,19,20,21]
Isometric	50	[5,22]
	15	[23]
	10	[24]
Dynamic	196	[25,26]
	100	[27,28,29,30,31,32]
	30	[33]
	20	[34,35]
	15	[36]
	<=10	[3,37,38,39,40]

**Table 2:** Partial Results of the N-way ANOVA on RMSE.

Factors	F-value	*p*-value
Freq. Range	5559.99	0.00
Action Type	308.18	0.00
Participant	53.96	0.00
Signal Type	74.78	0.00
Interpolation	1.53	0.22
Freq. Range *Interpolation	21.53	0.00
Action Type *Interpolation	0.07	0.93
Participant *Interpolation	0.03	1.00
Signal Type *Interpolation	0.29	0.75

Freq: Frequency; * indicates the interaction.

**Figure A1:**
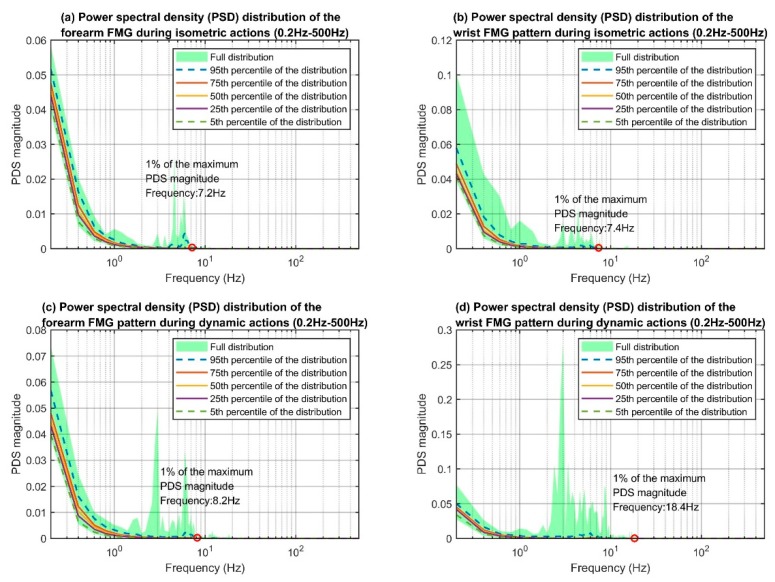
Maximum FMG power spectral density (PSD) distributions using Yule–Walker autoregressive method with order of 1000.

## Appendix A

### FMG Power Spectral Density Using Yule–Walker Auto-Regressive Algorithm

The PSD of FMG using Yule–Walker’s auto-regressive (AR) method is shown in Figure A1. For the AR method, the order of the model is a hyperparameter that should be tuned to reflect the signal content. Generally speaking, the higher the order, the higher the frequency resolution will be. However, if a very high order is used, the PSD may have many spectral peaks and its analysis will become very challenging. In this study, the AR order was varied within 10 to 5000 range. We found that the shape of PSD of the order of 1000 was not substantially different from the ones obtained from 500 or 5000. Therefore, we presented the PSD using an order of 1000 in the figure.

As observed in Figure A1, the PSD obtained using AR has a similar trend to that obtained using FFT (see Figure A1 and Figure 8). The PSD values initially decrease but then increase to around 5–7 Hz. However, when following the same steps and criteria to identify the minimum requirements for sampling frequencies of FMG signals as explained in Section 2, the PSD using AR resulted in values that were much lower than those identified using the FFT method. A detailed discussion of the discrepancies between the PSD using the FFT and the AR methods is out of the scope of this study but is a topic for future work. Overall, for a conservative measure, the results presented in the main manuscript remain valid for selecting the minimum sampling frequency for FMG signals.

## References

[1] Castellini C., Artemiadis P., Wininger M., Ajoudani A., Alimusaj M., Bicchi A., Caputo B., Craelius W., Dosen S., Englehart K. (2014). Proceedings of the first workshop on Peripheral Machine Interfaces: Going beyond traditional surface electromyography. Front. Neurorobot..

[2] Wininger M. (2008). Pressure signature of forearm as predictor of grip force. J. Rehabil. Res. Dev..

[3] Xiao Z.G., Menon C. (2017). Counting Grasping Action Using Force Myography: An Exploratory Study With Healthy Individuals. JMIR Rehabil. Assist. Technol..

[4] Ahmadizadeh C., Merhi L.-K., Pousett B., Sangha S., Menon C. (2017). Toward Intuitive Prosthetic Control: Solving Common Issues Using Force Myography, Surface Electromyography, and Pattern Recognition in a Pilot Case Study. IEEE Robot. Autom. Mag..

[5] Ravindra V., Castellini C. (2014). A Comparative Analysis of Three Non-Invasive Human-machine Interfaces for the Disabled. Front. Neurorobot..

[6] Lucaccini L.F., Kaiser P.K., Lyman J. (1966). The French electric hand: some observations and conclusions. Bull. Prosthet. Res..

[7] Phillips S.L., Craelius W. (2005). Residual kinetic imaging: A versatile interface for prosthetic control. Robotica.

[8] Li N., Yang D., Jiang L., Liu H., Cai H. (2012). Combined Use of FSR Sensor Array and SVM Classifier for Finger Motion Recognition Based on Pressure Distribution Map. J. Bionic Eng..

[9] Xiao Z.G., Menon C. (2017). Performance of Forearm FMG and sEMG for Estimating Elbow, Forearm and Wrist Positions. J. Bionic Eng..

[10] Xiao Z.G., Menon C. (2014). Towards the development of a wearable feedback system for monitoring the activities of the upper-extremities. J. Neuroeng. Rehabil..

[11] Dementyev A., Paradiso J.A. (2014). WristFlex. Proceedings of the 27th Annual ACM Symposium on User Interface Software and Technology.

[12] Jiang X., Xiao Z.G., Menon C. (2018). Virtual grasps recognition using fusion of Leap Motion and force myography. Virtual Real..

[13] Ha N., Withanachchi G.P., Yihun Y. Force Myography Signal-Based Hand Gesture Classification for the Implementation of Real-Time Control System to a Prosthetic Hand. Proceedings of the 2018 Design of Medical Devices Conference.

[14] Radmand A., Scheme E., Englehart K. (2016). High-density force myography: A possible alternative for upper-limb prosthetic control. J. Rehabil. Res. Dev..

[15] Radmand A., Scheme E., Englehard K. High-resolution muscle pressure mapping for upper-limb prosthetic control. Proceedings of the MEC—Myoelectric Control Symposium.

[16] Jiang X., Merhi L.-K., Menon C. (2017). Force Exertion Affects Grasp Classification Using Force Myography. IEEE Trans. Hum. -Mach. Syst..

[17] Ghataurah J., Ferigo D., Merhi L.K., Pousett B., Menon C. A Multi-sensor Approach for Biomimetic Control of a Robotic Prosthetic Hand. Proceedings of the 5th International Work-Conference (IWBBIO 2017).

[18] Chengani R., Delva M.L., Sakr M., Menon C. Pilot study on strategies in sensor placement for robust hand/wrist gesture classification based on movement related changes in forearm volume. Proceedings of the 2016 IEEE Healthcare Innovation Point-of-Care Technologies Conference (HI-POCT).

[19] Jiang X., Chu H.T., Xiao Z.G., Merhi L.-K., Menon C. Ankle positions classification using force myography: An exploratory investigation. Proceedings of the 2016 IEEE Healthcare Innovation Point-Of-Care Technologies Conference (HI-POCT).

[20] Cho E., Chen R., Merhi L.-K., Xiao Z., Pousett B., Menon C. (2016). Force Myography to Control Robotic Upper Extremity Prostheses: A Feasibility Study. Front. Bioeng. Biotechnol..

[21] Delva M.L., Sakr M., Chegani R.S., Khoshnam M., Menon C. Investigation into the Potential to Create a Force Myography-based Smart-home Controller for Aging Populations. Proceedings of the 7th IEEE International Conference on Biomedical Robotics and Biomechatronics (Biorob).

[22] Castellini C., Ravindra V. A wearable low-cost device based upon Force-Sensing Resistors to detect single-finger forces. Proceedings of the 5th IEEE RAS/EMBS International Conference on Biomedical Robotics and Biomechatronics.

[23] Belyea A.T., Englehart K.B., Scheme E.J. (2018). A proportional control scheme for high density force myography. J. Neural Eng..

[24] Sakr M., Menon C. On the estimation of isometric wrist/forearm torque about three axes using Force Myography. Proceedings of the 2016 6th IEEE International Conference on Biomedical Robotics and Biomechatronics (BioRob).

[25] Nowak M., Eiband T., Castellini C. Multi-modal myocontrol: Testing combined force- and electromyography. Proceedings of the 2017 International Conference on Rehabilitation Robotics (ICORR).

[26] Connan M., Ruiz Ramírez E., Vodermayer B., Castellini C. (2016). Assessment of a Wearable Force- and Electromyography Device and Comparison of the Related Signals for Myocontrol. Front. Neurorobot..

[27] Fang P., Ma X., Li X., Qiu X., Gerhard R., Zhang X., Li G. (2017). Fabrication, structure characterization, and performance testing of piezoelectret-film sensors for recording body motion. IEEE Sens. J..

[28] Li X., Zhuo Q., Zhang X., Samuel O.W., Xia Z., Zhang X., Fang P., Li G. FMG-based body motion registration using piezoelectret sensors. Proceedings of the 2016 38th Annual International Conference of the IEEE Engineering in Medicine and Biology Society (EMBC).

[29] Kamei Y., Okada S. Classification of forearm and finger motions using electromyogram and arm-shape-changes. Proceedings of the 2016 38th Annual International Conference of the IEEE Engineering in Medicine and Biology Society (EMBC).

[30] Yungher D., Craelius W. (2012). Improving fine motor function after brain injury using gesture recognition biofeedback. Disabil. Rehabil. Assist. Technol..

[31] Lukowicz P., Hanser F., Szubski C., Schobersberger W., Fishkin K.P., Schiele B., Nixon P., Quigley A. (2006). Detecting and Interpreting Muscle Activity with Wearable Force Sensors. Lecture Notes in Computer Science (including subseries Lecture Notes in Artificial Intelligence and Lecture Notes in Bioinformatics).

[32] Godiyal A.K., Mondal M., Joshi S.D., Joshi D. (2018). Force Myography Based Novel Strategy for Locomotion Classification. IEEE Trans. Hum. -Mach. Syst..

[33] Kadkhodayan A., Jiang X., Menon C. (2016). Continuous Prediction of Finger Movements Using Force Myography. J. Med. Biol. Eng..

[34] Sadarangani G.P., Jiang X., Simpson L.A., Eng J.J., Menon C. (2017). Force Myography for Monitoring Grasping in Individuals with Stroke with Mild to Moderate Upper-Extremity Impairments: A Preliminary Investigation in a Controlled Environment. Front. Bioeng. Biotechnol..

[35] Ogris G., Kreil M., Lukowicz P. (2007). Using FSR based muscule activity monitoring to recognize manipulative arm gestures. Proceedings of the 2007 11th IEEE International Symposium on Wearable Computers.

[36] Sadeghi Chegani R., Menon C. (2018). Regressing grasping using force myography: An exploratory study. Biomed. Eng. Online.

[37] Ferigo D., Merhi L.-K., Pousett B., Xiao Z.G., Menon C. (2017). A Case Study of a Force-myography Controlled Bionic Hand Mitigating Limb Position Effect. J. Bionic Eng..

[38] Sadarangani G., Menon C. A wearable sensor system for rehabilitation apllications. Proceedings of the 2015 IEEE International Conference on Rehabilitation Robotics (ICORR).

[39] Amft O., Troster G., Lukowicz P., Schuster C. Sensing Muscle Activities with Body-Worn Sensors. Proceedings of the International Workshop on Wearable and Implantable Body Sensor Networks (BSN’06).

[40] Sakr M., Menon C. Exploratory Evaluation of the Force Myography ( FMG ) Signals Usage for Admittance Control of a Linear Actuator. Proceedings of the 2018 7th IEEE International Conference on Biomedical Robotics and Biomechatronics (Biorob).

[41] Freund H.-J., Budingen H.J. (1978). The relationship between speed and amplitude of the fastest voluntary contractions of human arm muscles. Exp. Brain Res..

[42] FSR 400 Series Data Sheet. https://cdn2.hubspot.net/hubfs/3899023/Interlinkelectronics%20November2017/Docs/Datasheet_FSR.pdf.

[43] Xiao Z.G. (2017). Detecting Upper Extremity Activity with Force Myography. Ph.D. Thesis.

[44] Liu J., Feng F., Nakamura Y.C., Pollard N.S. A taxonomy of everyday grasps in action. Proceedings of the 2014 IEEE-RAS International Conference on Humanoid Robots.

[45] Zheng J.Z., De La Rosa S., Dollar A.M. An investigation of grasp type and frequency in daily household and machine shop tasks. Proceedings of the 2011 IEEE International Conference on Robotics and Automation.

[46] Bullock I.M., Dollar A.M. Classifying human manipulation behavior. Proceedings of the 2011 IEEE International Conference on Rehabilitation Robotics.

[47] Vergara M., Sancho-Bru J.L., Gracia-Ibáñez V., Pérez-González A. (2014). An introductory study of common grasps used by adults during performance of activities of daily living. J. Hand Ther..

[48] Kato A., Matsumoto Y., Kato R., Kobayashi Y., Yokoi H., Fujie M.G., Sugano S. (2018). Estimating wrist joint angle with limited skin deformation information. J. Biomech. Sci. Eng..

[49] Xiao Z.G., Menon C. (2019). Does force myography recorded at the wrist correlate to resistance load levels during bicep curls?. J. Biomech..

[50] Lee D.L., McLoone H., Dennerlein J.T. (2008). Observed finger behaviour during computer mouse use. Appl. Ergon..

[51] Feng P., Bresler Y. Spectrum-blind minimum-rate sampling and reconstruction of multiband signals. Proceedings of the 1996 IEEE International Conference on Acoustics, Speech, and Signal Processing Conference Proceedings.

[52] Kuboyama N., Nabetani T., Shibuya K.-I., Machida K., Ogaki T. (2004). The effect of maximal finger tapping on cerebral activation. J. Physiol. Anthropol. Appl. Hum. Sci..

[53] Morrison S., Hong S.L., Newell K.M. (2009). Upper frequency limits of bilateral coordination patterns. Neurosci. Lett..

[54] Jiang X., Merhi L.K., Xiao Z.G., Menon C. (2017). Exploration of Force Myography and surface Electromyography in hand gesture classification. Med. Eng. Phys..

[55] Most Drumbeats in a Minute Using Drumsticks. http://www.guinnessworldrecords.com/world-records/most-drumbeats-in-a-minute-using-drumsticks.

